# Predictors of paravalvular aortic regurgitation after surgery for Behcet’s disease-related severe aortic regurgitation

**DOI:** 10.1186/s13023-019-1083-8

**Published:** 2019-06-10

**Authors:** Hong-Mi Choi, Hyung-Kwan Kim, Sung-Ji Park, Hyun-Jung Lee, Yeonyee E. Yoon, Jun-Bean Park, Yong-Jin Kim, Goo-Young Cho, In-Chang Hwang, Dae-Won Sohn, Jae K. Oh

**Affiliations:** 1 Cardiovascular Center, Seoul National University Bundang Hospital, Seoul National University College of Medicine, Seongnam, Gyeonggi-do South Korea; 2 Division of Cardiology, Cardiovascular Center, Department of Internal Medicine, Seoul National University College of Medicine, Seoul, South Korea; 3 Division of Cardiology, Department of Medicine, Cardiovascular Imaging Center, Heart Vascular Stroke Institute, Samsung Medical Center, Sungkyunkwan University School of Medicine, Seoul, South Korea; 4 Department of Cardiovascular Diseases, Mayo Clinic, Rochester, MN USA

**Keywords:** Behcet’s disease, Aortic regurgitation, Paravalvular leakage, Aortic valve surgery

## Abstract

**Background:**

Behcet’s disease (BD)-related aortic regurgitation (AR) is known to be associated with paravalvular leakage (PVL) after successful aortic valve (AV) surgery. This study aimed to determine predictors of PVL after successful AV surgery in BD patients. We retrospectively collected data of 35 patients (42.1 ± 9.1 years, 27 men) who underwent surgery for severe BD-related AR at two tertiary centers. The diagnosis was established based on echocardiographic, surgical, and/or pathological findings in conjunction with the International Study Group criteria for BD. A total of 76 cases of AV surgery in 35 patients were analyzed.

**Results:**

A median follow-up duration was 8.0 years (interquartile range, 5.4–14.3 years). PVL developed in 18 patients (51.4%) within 2 years after the first surgery. Six patients who met the diagnostic criteria for BD did not develop PVL, in whom 5 patients took immunosuppressive therapy (IST). However, 4 of 9 patients (44.4%) who did not meet the diagnostic criteria developed PVL, in whom four (44.4%) patients took IST. On multivariable analysis, postoperative IST and concomitant aortic root replacement (ARR) were two independent predictors for less PVL development (HR 0.38, 95% CI 0.17–0.89, *p* = 0.025 for postoperative IST; HR 0.17, 95% CI 0.08–0.36, *p* < 0.001 for concomitant ARR). Preoperative IST use did not determine PVL development (*p* = 0.75).

**Conclusions:**

Postoperative, but not preoperative, IST and concomitant ARR were independent predictors of less development of PVL. Special attention is required for early diagnosis BD-related AR, especially in patients not satisfying the current diagnostic criteria.

**Electronic supplementary material:**

The online version of this article (10.1186/s13023-019-1083-8) contains supplementary material, which is available to authorized users.

## Background

Behcet’s disease (BD) is a chronic systemic inflammatory disorder of unknown etiology, characterized by recurrent orogenital ulcers and other organ involvement such as skin and eyes. Cardiac involvement in BD is uncommon but is associated with life-threatening complications [1–6]. Among various manifestations of cardiac BD, aortic regurgitation (AR) is the most serious complication, because early diagnosis and surgical treatment are challenging [3, 4]. The fragility of aortic structures and tissue inflammation in BD make surgical treatment of AR more difficult, frequently resulting in paravalvular leakage (PVL), pseudoaneurysm formation, and even death [3–5, 7–9]. Prognosticators have been suggested, however, the number of patients included in earlier studies was too small to draw robust conclusions. This study aimed to determine the predictors of PVL after corrective aortic valve (AV) surgery for BD-related AR at two large tertiary university hospitals.

## Methods

### Patient selection

We meticulously reviewed medical records of 636 patients who underwent corrective AV surgery for severe AR at two tertiary centers (354 patients at Seoul National University Hospital [SNUH] from 2008 to 2016, and 282 patients at Samsung Medical Center [SMC] from 1995 to 2016) and selected BD-related AR patients (Additional file 1: Figure S1). AV surgeries included AV repair, AV replacement (AVR), and composite graft replacement of the AV, aortic root, and ascending aorta (i.e., Bentall procedure).

**Fig. 1:**
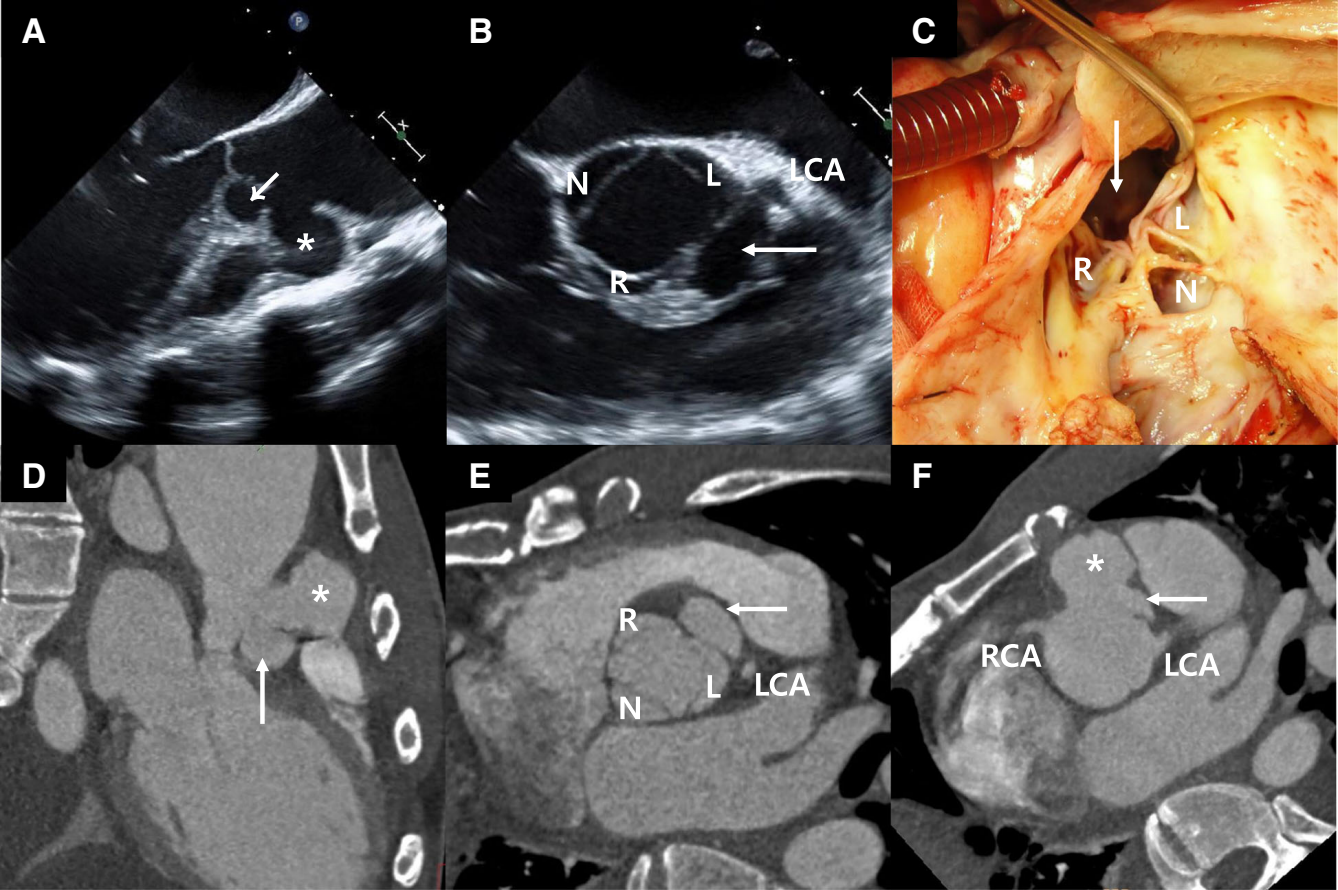
Representative case of Behcet’s disease-related aortic regurgitation. A 36-year-old male presented with Behcet’s disease-related severe aortic regurgitation. Pseudoaneurysm (asterisk) and interventricular septal dissection (arrow) between the right and left coronary cusps were clearly shown on transesophageal echocardiography (**a**, **b**), in the surgical field (**c**) and on computed tomography (**d-f**). R, right coronary cusp; L, left coronary cusp; N, non-coronary cusp; LCA, left coronary artery; RCA, right coronary artery


Diagnosis of BD-related AR was preoperatively and/or postoperatively established based on clinical, echocardiographic features, and pathology of surgical specimens. Characteristic echocardiographic features of AV involvement in BD included 1) aneurysmal change of AV cusp, 2) pseudoaneurysm of aortic root or ascending aorta, and 3) dissection of the interventricular septum, as previously suggested (Fig. 1, Additional file 2: Video S1, Additional file 3: Video S2, Additional file 4:Video S3) [3–5, 8, 10]. Aneurysmal change of AV cusp is defined as a primary ectatic change of AV cusp with redundant motion, resulting in AV malcoaptation with severe AR.

**Additional file 2:** **Video S1.** Representative, characteristic echocardiographic images in patients with BD-related severe AR. A 45-year-old man showed an aneurysmal change of non-coronary cusp of aortic valve, resulting in severe aortic regurgitation. (AVI 910 kb)



**Additional file 3:** **Video S2.** Representative, characteristic echocardiographic images in patients with BD-related severe AR. A 46-year-old woman showed interventricular septal dissection and additional destructive aneurysmal changes. (AVI 837 kb)



**Additional file 4:** **Video S3.** Representative, characteristic echocardiographic images in patients with BD-related severe AR. A 46-year-old woman showed interventricular septal dissection and additional destructive aneurysmal changes. (AVI 840 kb)


Because it was reported that BD patients with severe AR or other vascular involvement do not fully meet the ISG criteria in many cases [3, 4, 8, 11–13], 35 patients were categorized based on the International Study Group (ISG) criteria [14] to evaluate the sensitivity of the conventional diagnostic criteria for BD in patients with BD-related severe AR. “Definite BD” was defined if a patient showed recurrent oral ulcers and at least two of the following features; recurrent genital ulcers, ocular lesions, skin lesions, and a positive pathergy test result. “Suspected BD” was defined if a patient showed recurrent oral aphthous ulcers plus one additional organ involvement. Patients with recurrent oral ulcer only and those who did not meet any diagnostic criterion were classified separately. To confirm the diagnosis in these patients, great effort was particularly made to reach a consensus on the diagnosis of BD among cardiologists, rheumatologists, cardiac pathologists, and cardiac surgeons. In addition, for patients who previously underwent AV surgery elsewhere, external echocardiographic images, medical records at the time of the first AV surgery including surgical records and pathological findings were thoroughly reviewed. Patients with features suggesting infective endocarditis such as vegetation, positive blood cultures at the first surgery, and positive Gram staining in the surgical specimen were systematically excluded.

Using the above criteria, we found 20 patients with BD-related AR, with an overall prevalence rate of 3.1% (20/636, 2.8% in SNUH and 3.5% in SMC) among the patients who underwent AV surgery due to severe AR. On top of that, we also collected follow-up data of 15 patients with BD-related AR that were previously published elsewhere [4]. Therefore, a total of 35 patients with severe BD-related AR were finally analyzed in this report (Additional file 1: Figure S1). This study was approved by the institutional review board of each hospital. The need for informed consent was waived owing to the retrospective nature of this study.

### Patient management

The type of surgery performed was determined at the discretion of the attending cardiac surgeons. Surgical method used was previously described in detail [5]. Pre- or postoperative immunosuppressive therapy (IST) was mainly guided by rheumatologists. Although standard IST regimen for BD-related AR has not been established yet [15], intravenous corticosteroid was a medication of choice prescribed before surgery, and low dose of oral corticosteroid with/without azathioprine was prescribed after surgery in most patients.

### Data procurement

Clinical records of all patients undergoing AV surgery, including medical charts and preoperative echocardiographic findings, were retrospectively reviewed. Surgical findings, pathological findings for the surgical specimen, and follow-up data on PVL development, additional surgical procedure, pacemaker implantation for complete atrioventricular block (CAVB), and death were also obtained. According to earlier studies on BD-related AR [4], most BD-related PVL after index surgery was observed within 2 years after surgery. Thus, PVL detected within 2 years after each surgery was considered BD-related PVL.

### Statistical analysis

Categorical variables were expressed as numbers (percentages), and continuous variables as median with interquartile range or mean ± standard deviation, as appropriate. We used Cox proportional hazards model to find factors predicting BD-related PVL, and the hazard ratio (HR) was adjusted for clinical risk factors showing significant associations (*p* < 0.1) with outcomes in the univariate analysis. The final model was further adjusted for intra-cluster correlation because some patients underwent multiple AV surgeries for recurrent PVL. Kaplan-Meier curves were generated to estimate the survival function of time-to-events according to surgical technique and IST use. Differences in the curves were compared using the log-rank test. A two-sided *p* value < 0.05 was considered statistically significant. All statistical analyses were conducted using R software version 3.4.2.

## Results

### Baseline characteristics and diagnosis of BD

The baseline characteristics of the 35 patients are summarized in Table 1. BD was diagnosed according to the ISG criteria prior to the first surgery and was well under control by IST in four patients (11.4%). In nine patients (25.7%), the ISG criteria was not met prior to the first surgery, but echocardiographic and surgical findings were highly suggestive of BD. The remaining 22 patients (62.9%) were finally diagnosed with severe BD-related AR after prosthetic valve failure developed, given the surgical and pathological findings.

**Table 1:** Baseline characteristics and outcomes

	All patients (*N* = 35)
Baseline	
Age at the 1^st^ surgery (years)	42.1 ± 9.1 (23, 60)
Male, n (%)	27 (77.1%)
ESR (mm/hr)	31.9 ± 36.8
CRP (mg/dL)	1.1 ± 1.8
Clinical manifestations of BD	
Recurrent oral ulcer	32 (91.4%)
Genital ulcer	9 (25.7%)
Eye involvement	1 (2.9%)
Skin lesion	21 (60.0%)
Positive Pathergy test	2 (5.7%)
Fulfillment of ISG criteria	
Definite	6 (17.1%)
Suspected	20 (57.1%)
Oral ulcer only	6 (17.1%)
None	3 (8.6%)
Outcomes	
Death	16 (45.7%)
Cardiac death	11 (31.4%)
Heart transplantation	1 (2.9%)
Complete atrioventricular block	17 (48.6%)
Number of open heart surgeries	2.4 ± 1.1
More than 2 open heart surgeries	28 (80.0%)
AV paravalvular leakage within 2 years	18 (51.4%)

Values are mean ± SD (minimum value, maximum value) or n (%)

*ESR* erythrocyte sediment rate, *CRP* C-reactive protein, *BD* Behcet’s disease, *ISG* International study group, *AV* aortic valve

Throughout the disease course, BD could be confirmed in six (17.1%) patients based on the ISG criteria, including four patients already diagnosed with BD prior to the first surgery (definite BD group). Twenty (57.1%) patients were classified into the suspected BD group. Six patients (17.1%) complained of recurrent oral ulcers only, and the remaining three (8.6%) did not meet any component of the ISG criteria. On the other hand, 26 (74.3%) of 35 patients could be diagnosed with BD based on the International Criteria for Behcet’s Disease (ICBD) criteria [16].

More than half of patients (19 [57.1%]) initially complained of exertional dyspnea and/or orthopnea, with consequent development of chest pain and palpitation (6 [17.1%] for both symptoms). Syncope and low-grade fever were the presenting symptoms in four (11.4%) and one (2.9%), respectively. Blood cultures were negative in all 35 patients. Only 2 patients had an ascending aorta diameter more than 45 mm on the initial computed tomographic angiography or echocardiography, in both of whom aortic root replacement (ARR) was performed along with AV replacement. Other vascular involvement was not found in any patient.

### Long-term outcomes and PVL development (Table 1*,* Fig. 2)

**Fig. 2:**
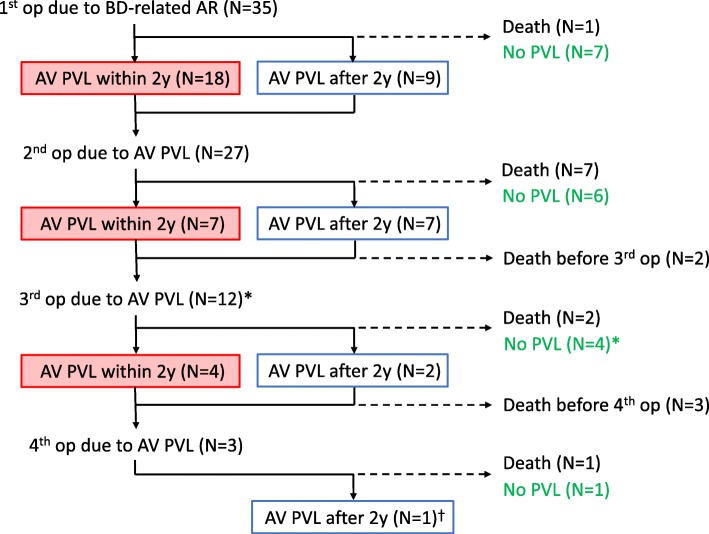
Summary of clinical outcomes of 35 patients with Behcet’s disease-related aortic regurgitation. Of the 35 patients who underwent aortic valve (AV) surgery, 18 developed paravalvular leakage (PVL) within 2 years after surgery. One patient died, and 7 patients survived without PVL. Along with 9 patients who had PVL at 2 years after surgery, 27 patients underwent the second surgery for PVL. Two patients died before the third surgery, and 7 developed PVL within 2 years. Of the 12 patients who underwent the third surgery, 4 developed PVL within 2 years after surgery and 2 developed PVL 2 years after surgery. Three patients died before the fourth surgery and 3 patients underwent the fourth surgery. One patient died, and 1 survived without PVL, and 1 developed PVL after 2 years but survived without surgery. A total of 76 AV surgeries were performed, and 29 PVLs developed within 2 years after AV surgery. * Including one heart transplantation. † The patient has survived with medical treatment 10 years after the fourth surgery

All patients were followed up until June 2017, with a median follow-up duration of 8.0 years (minimum 255 days, maximum 23.9 years; interquartile range, 5.4–14.3 years). Sixteen patients (45.7%) died, and one (2.9%) underwent heart transplantation. Causes of non-cardiac death were cancer progression (*n* = 2), uncontrolled colitis (*n* = 1), stroke (*n* = 1), and unknown (*n* = 1). Cardiac death occurred in 11 patients (31.4%), of whom, three died of immediate postoperative complications (two, prosthetic valve endocarditis; three, in-hospital cardiac arrest; one, heart failure progression), and two, sudden death outside the hospital. Of the three patients who died of cardiac arrest in the hospital, two died preoperatively during admission for PVL surgery, and one during admission for pacemaker implantation for CAVB (Fig. 2).

PVL at the prosthetic AV developed in 18 patients (51.4%) within 2 years after the first surgery for severe AR. All six patients who met the ISG criteria (i.e., definite BD group) did not experience PVL, in whom 5 patients took IST. In contrast, PVL developed in 14 of 20 patients (70%) of the suspected BD group in whom only two (10%) took IST, and PVL developed in 4 of 9 patients (44.4%) who did not meet the ISG criteria in whom four (44.4%) patients took IST. In summary, none of the patients in the definite BD group developed PVL, whereas a substantial number of patients who were not included in the definite BD group had PVL (Additional file 1: Figure S2).

**Fig. 3:**
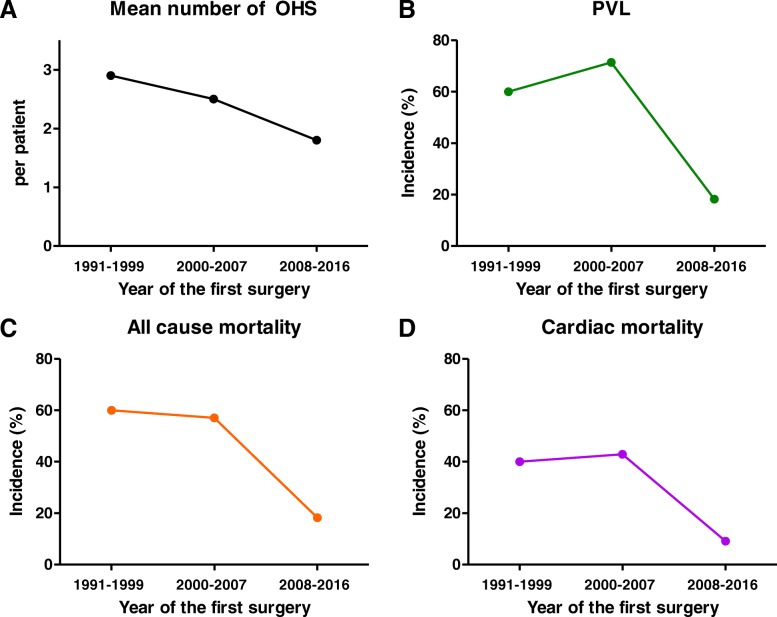
Temporal trends of outcomes according to the year of the first surgery. With time, there was a decreasing trend in (**a**) mean number of open heart surgeries (OHS) per patient, (**b**) paravalvular leakage (PVL) development, (**c**) all-cause mortality, and (**d**) cardiac mortality. Please note that clinical outcome has markedly improved since 2008, when unique echocardiographic findings suggesting severe Behcet’s disease-related aortic regurgitation were clinically introduced

As shown in Fig. 3, interestingly, the number of open heart surgeries (OHS) per patient, all-cause mortality, cardiac mortality, and the incidence of PVL at the prosthetic AV tended to decrease after the clinical application of characteristic echocardiographic findings [3–5, 8] to diagnose severe BD-related AR.

Overall, 83 OHSs were performed (mean, 2.4 ± 1.1; minimum 1, maximum 5) during follow-up; 76, AV surgeries; 3, pseudoaneurysm repairs; 3, isolated mitral valve replacements; and 1, heart transplantation. Of note, 28 patients (80%) underwent OHSs more than two times. We could not conclude that the three isolated mitral valve replacements were related to BD, based on the available data; however, BD might be a plausible cause of at least two surgeries, because PVL was the major pathological finding.

### Predictors of PVL in 76 AV surgeries

**Fig. 4:**
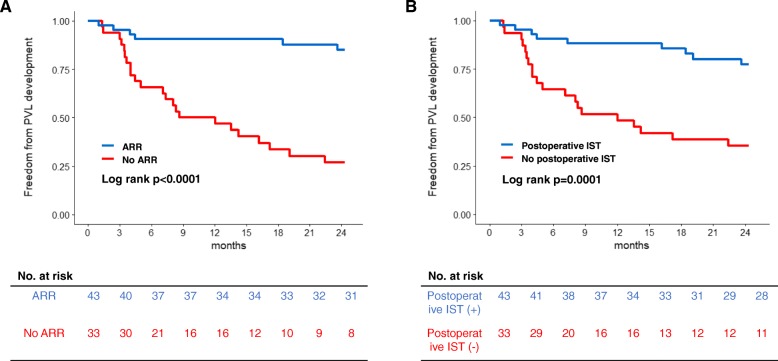
Kaplan-Meier estimates of freedom from paravalvular leakage (PVL) stratified by (**a**) aortic root replacement (ARR) and (**b**) postoperative immunosuppressive therapy (IST). The incidence of PVL was significantly higher in patients who did not undergo postoperative IST or concomitant ARR

A total of 76 AV surgeries were analyzed to estimate the risk of PVL for each AV surgery. PVL at the prosthetic AV developed after 29 AV surgeries. Univariate Cox proportional hazards analysis showed that female sex, preoperative and postoperative ISTs, and concomitant ARR were associated with less PVL development. Notably, preoperative erythrocyte sedimentation rate was not a predictor of PVL in the univariate analysis. In the multivariate analysis, concomitant ARR and use of postoperative IST were two independent predictors of less PVL development (HR 0.17, 95% CI 0.08–0.36, *p* < 0.001 for concomitant ARR; HR 0.38, 95% CI 0.17–0.89 *p* = 0.025 for postoperative IST) (Table 2). We also performed sensitivity analysis to confirm the role of preoperative IST in the prevention of PVL development, because all but one patient undergoing preoperative IST took postoperative IST. Of 76 AV surgeries, postoperative IST was used in 43 cases, among which preoperative IST was conducted in 32 cases (74.4%). Kaplan-Meier curve for PVL development showed that there was no difference in PVL development irrespective of preoperative IST use (HR 1.27, 95% CI 0.2–5.45, *p* = 0.752) (Additional file 1: Figure S3). Kaplan-Meier curves for PVL development stratified by concomitant ARR and postoperative IST and are illustrated in Fig. 4*.* Patients who did not undergo postoperative IST or concomitant ARR showed a significantly higher incidence of PVL.

**Table 2:** Cox proportional hazard regression model for predicting paravalvular leakage development in 76 aortic valve surgeries

	Univariable analysis			Multivariable analysis		
	HR	95% CI	*P* value	HR	95% CI	*P* value
Age at the time of surgery (years)	0.98	0.95–1.01	0.147	1.01	0.98–1.05	0.463
Female sex	0.13	0.02–0.91	0.040	0.09	0.01–0.79	0.030
ESR (mm/hr)	1.00	0.98–1.02	0.905			
Number of OHS performed	0.79	0.47–1.34	0.381			
Use of preoperative IST	0.40	0.17–0.95	0.038			
Use of postoperative IST	0.23	0.10–0.53	< 0.001	0.38	0.17–0.89	0.025
Concomitant ARR	0.13	0.05–0.33	< 0.001	0.17	0.08–0.36	< 0.001

*HR* Hazard ratio, *CI* confidence interval, *ESR* erythrocyte sediment rate, *OHS* open heart surgery, *IST* immunosuppressive therapy, *ARR* aortic root replacement

### CAVB complicated in BD-related AR

CAVB requiring permanent pacemaker implantation developed in 17 patients (48.6%). All patients underwent permanent pacemaker implantation, except 2 patients: one died of cardiac arrest before pacemaker implantation, and the other refused the procedure. Among the 17 patients requiring pacemaker implantation for CAVB, information on the AV cusp involved on the first preoperative echocardiography and/or the first surgical record could be obtained in 11 patients. Non-coronary cusp of AV was involved in 9 of 11 patients (81.8%).

## Discussion

The major findings of this study are summarized as follows: 1) The prevalence of severe BD-related AR is approximately 3.1% in all patients with severe AR of diverse etiologies requiring AV surgery. 2) The rate of complications such as PVL requiring repeat OHS, atrioventricular conduction disturbance, and death was not low. 3) Since the clinical introduction of pathognomonic echocardiographic findings suggesting severe BD-related AR, the incidence rate of PVL, the number of required repeated OHSs, and cardiac mortality tended to decrease. 4) BD-related PVL developed more frequently in patients not meeting the conventional ISG criteria. 5) Finally, concomitant ARR and postoperative IST were two independent predictors for less PVL development, whereas preoperative IST was not.

AR is a rare complication of BD [2–4, 8, 12, 17], and its pathophysiology has yet to be completely understood [17]. Although annular dilation and sinus of Valsalva pseudoaneurysm formation secondary to aortitis had been suggested [18], direct valvular inflammation was also proposed as a critical mechanism causing AR [2, 12]. Valvular inflammation could lead to aneurysmal changes in the AV cusp, pseudoaneurysm of aortic root, and mass-like lesions, all of which are currently considered pathognomonic echocardiographic findings of AR in BD (Additional files 2, 3 and 4: Video S1 S2 and S3) [3–5, 12].

Pathophysiology of recurrent PVL after corrective AV surgery is not completely understood in this population, although defective tissue-healing process due to inflammation was suggested as a main pathophysiology [19]. The frequency of early PVL after the first surgery in patients with BD-related AR was significantly higher (18 of 35 patients, 51.4%), compared to the rate of 2-year PVL (4 of 404 patients, 1.0%) reported in a previous study, where patients diagnosed with infective endocarditis who underwent AVR were included [20]. Thus, it is essential to find the appropriate management strategy (i.e., early diagnosis and appropriate perioperative treatment) to improve postoperative outcome. However, data has been insufficient, possibly owing to the rarity of the disease and difficulties in the diagnosis of BD-related AR prior to full-blown presentation of the ISG criteria.

Because BD has no pathognomonic single diagnostic test, ISG criteria have been used since 1990 [14]. Notwithstanding, there were multiple reports showing that the ISG criteria were not useful for the early diagnosis of BD-related AR [3, 4, 8, 11–13]. Consistent with earlier reports, we also observed that only 6 patients (17.1%) fulfilled the ISG criteria prior to the first AV surgery. Given poor sensitivity of the ISG criteria in diagnosing BD-related AR [3, 4, 7, 8, 11–13], diagnosis and treatment may be delayed, leading to poor prognosis. The absence of vascular manifestations in the ISG criteria may be one reason why the ISG criteria are considered less sensitive for the diagnosis of BD-related AR. Besides, the ISG criteria were originally suggested to elicit common consent for the diagnosis of BD [14]; thus, it is not suitable for the early detection of rare manifestations of BD on an individual basis. The ICBD introduced vascular and neurologic manifestations as additional criteria for the diagnosis of BD [16], and the application of this criteria in our study population could allow the diagnosis of BD in up to 26 patients (74.3%). Nevertheless, nine patients in our series were still not put on the diagnostic list of BD. Thus, we need to keep in mind that severe AR can be an initial manifestation of BD even in patients who do not meet the current diagnostic criteria. As the initial transthoracic echocardiography in all patients invariably showed pathognomonic findings reported in the literature [3–5, 8, 10], we believe that transthoracic echocardiography can play a critical role in the early diagnosis of BD-related AR, even in patients who do not meet the ISG or ICBD criteria [3, 4, 8], and should be used clinically as a single specific diagnostic test for the early diagnosis of BD-related AR. We also observed that patients who did not definitely meet the ISG criteria had a poorer 2-year PVL-free survival rate, whereas the definite BD group carried better prognosis. This may be related to the early establishment of IST, which was confirmed by the multivariate analysis in the current study. In fact, we observed that postoperative IST, but not preoperative IST, emerged as an independent factor preventing PVL development after multivariate analysis. Besides, as preoperative IST was used in almost every case in which postoperative IST was used, the direct comparison of efficacy between preoperative and postoperative IST was not fair. To avoid this statistical fallacy, we performed the sensitivity analysis (Additional file 1: Figure S3) and confirmed that postoperative IST is an independent predictor along with concomitant ARR. This observation can give comfort to cardiologists or rheumatologists who need to decide whether to proceed with AV surgery, because most patients with BD-related severe AR can present with acute heart failure and require urgent surgery without effective control of systemic inflammation. Therefore, it may not be acceptable to delay corrective AV surgery in patients with BD-related severe AR to control for aortitis or valvulitis. We should also remember the importance of concomitant ARR as an effective surgical option to decrease PVL in this population. As the pathergy test implies, exaggerated inflammatory response to the injury to the unhealthy aorta can be elicited in patients with active BD [19, 21]. Thus, suturing artificial prosthesis such as mechanical or bioprosthetic valve to the inflamed aortic root could lead to unsuccessful healing, resulting in early PVL due to dehiscence. Concomitant ARR at the time of AVR using premade valved conduits or homografts can be the best option to avoid prosthetic valve dehiscence.

CAVB is a well-known complication of BD-related AR. We found that its incidence was 48.6%, whereas the incidence of CAVB requiring pacemaker implantation after general AR surgery was reported to be 3.2% [22]. Because the atrioventricular node is located just below non-coronary cusp of AV [23], the inflammation of aortic root, esp. non-coronary cusp of AV, and its expansion to the neighboring atrioventricular node can be a potential explanation regarding high frequency of CAVB. This explanation is advocated by the observation that the non-coronary cusp of AV was involved in 9 of 11 patients (81.8%) in our series. In addition, development of interventricular septal dissection (Additional file 3: Video S2 and Additional file 4: Video S3) mechanically blocks the atrioventricular node conduction, resulting in CAVB.

In the past, less attention was paid to BD-related AR, mainly because of its rarity and absence of pathognomonic imaging findings. Since the introduction of characteristic echocardiographic findings [3–5, 8, 10], early diagnosis and early intervention have been possible, which is expected to have a favorable impact on outcomes. This is an important issue but is difficult to prove, because severe BD-related AR is a rare disease, and thus, a prospective study is extremely difficult to perform. Instead, we attempted to investigate the temporal trends of the incidence of PVL, number of required OHSs, and cardiac mortality by dividing the study population into three groups according to the time point when the first surgery was conducted. The decreasing trend of adverse events are shown in Fig. 3 suggesting that early detection and appropriate medical and surgical treatment should improve clinical outcomes.

Several limitations should be acknowledged. Firstly, only 35 patients with BD-related severe AR were found and analyzed, making the study susceptible to low statistical power and overfitting issue. However, BD-related AR is rare in prevalence, and to our knowledge, the present study included the largest population of patients with BD-related severe AR requiring AV surgery. Although we included 15 patients who were included in the previous study [4], the analysis of our study was completely different from the previous one. Secondly, nine patients in this study failed to fulfill the current ISG or ICBD criteria for a definite diagnosis of BD; hence, there might be an argument regarding the diagnostic accuracy in these patients. However, surgical and pathological findings for all patients showed characteristic histopathology suggesting AV involvement in BD, and thus, all relevant experts finally agreed on the diagnosis of BD. Thirdly, the institutions at which the study performed were tertiary referral centers, therefore the prevalence of the BD-related severe AR requiring AVR could be overestimated. Finally, we initially searched for patients with severe AR who underwent AV surgery; therefore, seriously ill patients who could not proceed with surgical intervention might not have been included in this study, leaving the possibility of underestimation of the prevalence of BD-related severe AR.

## Conclusions

In conclusion, BD-related severe AR requiring AV surgery is a rare but clinically important disease entity. Postoperative IST and concomitant ARR, but not preoperative IST, are independent predictors of less PVL development. A special attention is required to early diagnose BD-related AR, especially in patients who did not satisfy the ISG criteria. The active use of pathognomonic echocardiographic findings could facilitate early diagnosis.

## Additional files

Additional file 1: **Figure S1.** Schematic diagram showing the selection process of the patient population. **Figure S2.** Kaplan-Meier estimates of freedom from paravalvular leakage (PVL) stratified by satisfaction of the International Study Group (ISG) criteria. **Figure S3.** Kaplan-Meier estimates of freedom from paravalvular leakage (PVL) in patients who received postoperative immunosuppressive therapy (IST), stratified by the use of preoperative (preop) IST. (DOCX 378 kb)

## Abbreviations

AR: Aortic regurgitation
AV: Aortic valve
AVR: Aortic valve replacement
BD: Behcet’s disease
CAVB: Complete atrioventricular block
ICBD: International Criteria for Behcet's Disease
ISG: International Study Group
IST: Immunosuppressive therapy
OHS: Open heart surgery
PVL: Paravalvular leakage

## References

[1] James DG, Thomson A (1982). Recognition of the diverse cardiovascular manifestation in Behcet's disease. Am Heart J.

[2] Lee CW, Lee J, Lee WK (2002). Aortic valve involvement in Behcet's disease. A clinical study of 9 patients. Korean J Intern Med.

[3] Song JK, Jeong YH, Kang DH (2003). Echocardiographic and clinical characteristics of aortic regurgitation because of systemic vasculitis. J Am Soc Echocardiogr.

[4] Han JK, Kim HK, Kim YJ (2009). Behcet's disease as a frequently unrecognized cause of aortic regurgitation: suggestive and misleading echocardiography findings. J Am Soc Echocardiogr.

[5] Jeong DS, Kim KH, Kim JS, Ahn H (2009). Long-term experience of surgical treatment for aortic regurgitation attributable to Behcet's disease. Ann Thorac Surg.

[6] Geri G, Wechsler B, Thi Huong d L (2012). Spectrum of cardiac lesions in Behcet disease: a series of 52 patients and review of the literature. Medicine (Baltimore).

[7] Ahn JK, Kim H, Lee J (2009). Treatment outcomes in patients with non-infectious aortic valvulitis undergoing aortic valve replacement: implication for the treatment of aortic valve involvement in Behcet's disease. Rheumatol Int.

[8] Song JK, Kim MJ, Kim DH (2011). Factors determining outcomes of aortic valve surgery in patients with aortic regurgitation due to Behcet's disease: impact of preoperative echocardiographic features. J Am Soc Echocardiogr.

[9] Lakhanpal S, Tani K, Lie JT, Katoh K, Ishigatsubo Y, Ohokubo T (1985). Pathologic features of Behcet's syndrome: a review of Japanese autopsy registry data. Hum Pathol.

[10] Chikamori T, Doi YL, Yonezawa Y, Takata J, Kawamura M, Ozawa T (1990). Aortic regurgitation secondary to Behcet's disease. A case report and review of the literature. Eur Heart J.

[11] Tascilar K, Melikoglu M, Ugurlu S, Sut N, Caglar E, Yazici H (2014). Vascular involvement in Behcet's syndrome: a retrospective analysis of associations and the time course. Rheumatology (Oxford).

[12] Lee I, Park S, Hwang I (2008). Cardiac Behcet disease presenting as aortic valvulitis/aortitis or right heart inflammatory mass: a clinicopathologic study of 12 cases. Am J Surg Pathol.

[13] Kim JN, Kwak SG, Choe JY, Kim SK. The prevalence of Behcet's disease in Korea: data from Health Insurance Review and Assessment Service from 2011 to 2015. Clin Exp Rheumatol. 2017;35 Suppl 108(6):38–42. 28134076

[14] Criteria for diagnosis of Behcet's disease (1990). International study Group for Behcet's disease. Lancet..

[15] Hatemi G, Christensen R, Bang D (2018). 2018 update of the EULAR recommendations for the management of Behcet's syndrome. Ann Rheum Dis.

[16] International Team for the Revision of the International Criteria for Behcet's (2014). D. the international criteria for Behcet's disease (ICBD): a collaborative study of 27 countries on the sensitivity and specificity of the new criteria. J Eur Acad Dermatol Venereol.

[17] Tsui KL, Lee KW, Chan WK (2004). Behcet's aortitis and aortic regurgitation: a report of two cases. J Am Soc Echocardiogr.

[18] Tai YT, Fong PC, Ng WF (1991). Diffuse aortitis complicating Behcet's disease leading to severe aortic regurgitation. Cardiology..

[19] Calamia KT, Schirmer M, Melikoglu M (2005). Major vessel involvement in Behcet disease. Curr Opin Rheumatol.

[20] Schaff HV, Carrel TP, Jamieson WR (2002). Paravalvular leak and other events in silzone-coated mechanical heart valves: a report from AVERT. Ann Thorac Surg.

[21] Inaloz HS, Evereklioglu C, Unal B, Kirtak N, Eralp A, Inaloz SS (2004). The significance of immunohistochemistry in the skin pathergy reaction of patients with Behcet's syndrome. J Eur Acad Dermatol Venereol.

[22] Limongelli G, Ducceschi V, D'Andrea A (2003). Risk factors for pacemaker implantation following aortic valve replacement: a single Centre experience. Heart..

[23] Hahn RT, Nicoara A, Kapadia S, Svensson L, Martin R (2018). Echocardiographic imaging for Transcatheter aortic valve replacement. J Am Soc Echocardiogr.

