# Supplementary material for: Predictors of paravalvular aortic regurgitation after surgery for Behcet’s disease-related severe aortic regurgitation
Source: Orphanet J Rare Dis. 2019 Jun 10;14:132. doi: 10.1186/s13023-019-1083-8 (PMC6558675; doi:10.1186/s13023-019-1083-8)

**Figure S1. Schematic diagram showing the selection process of the patient population**

OHS, open heart surgery; AR, aortic regurgitation; BD, Behcet’s disease.

**
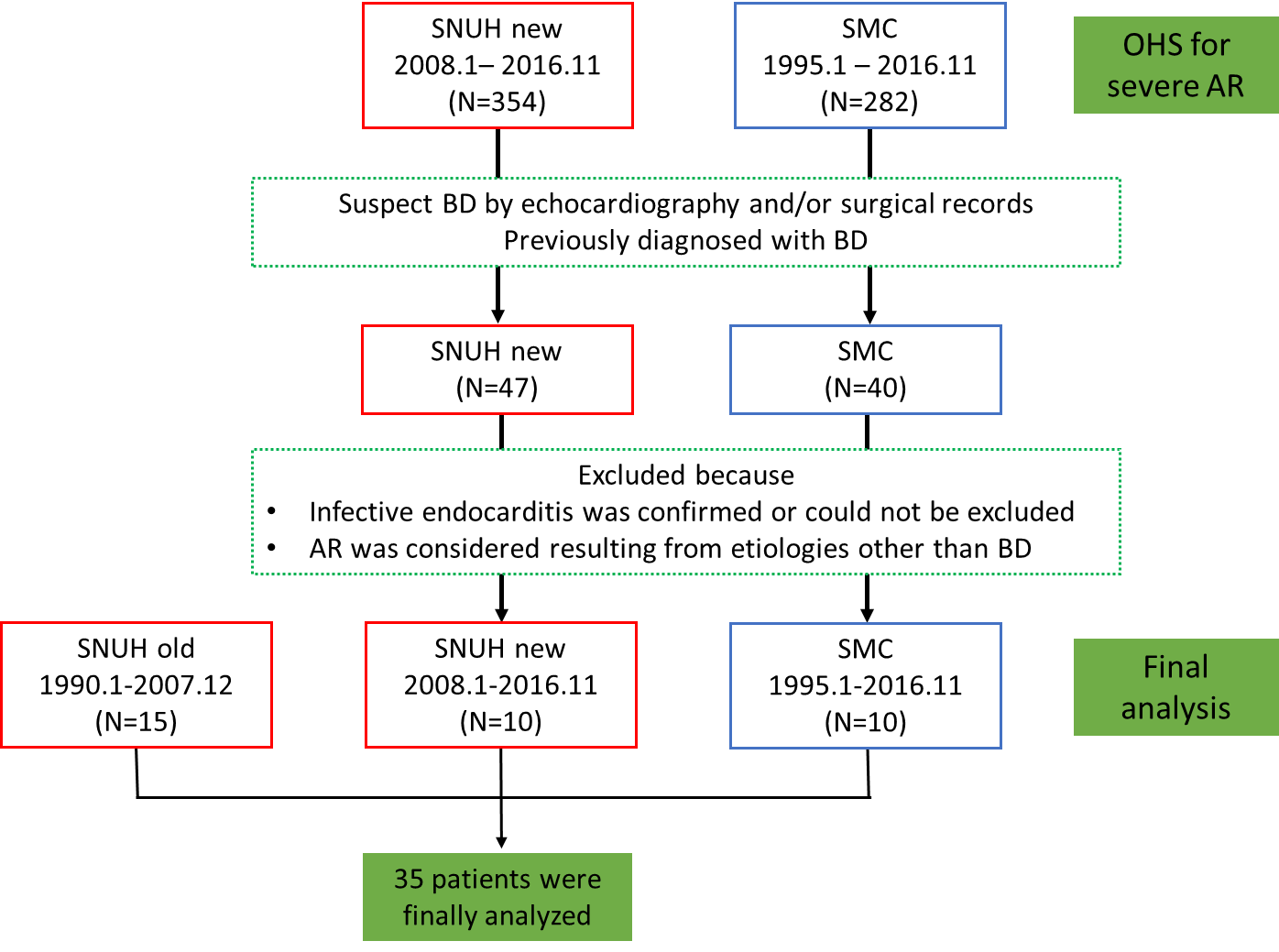
**

**Figure S2. Kaplan-Meier estimates of freedom from paravalvular leakage (PVL) stratified by satisfaction of the International Study Group (ISG) criteria**

The PVL developed more frequently in patients who did not meet the ISG criteria, whereas no patients who definitely met the ISG criteria had PVL. *BD*, Behcet’s disease


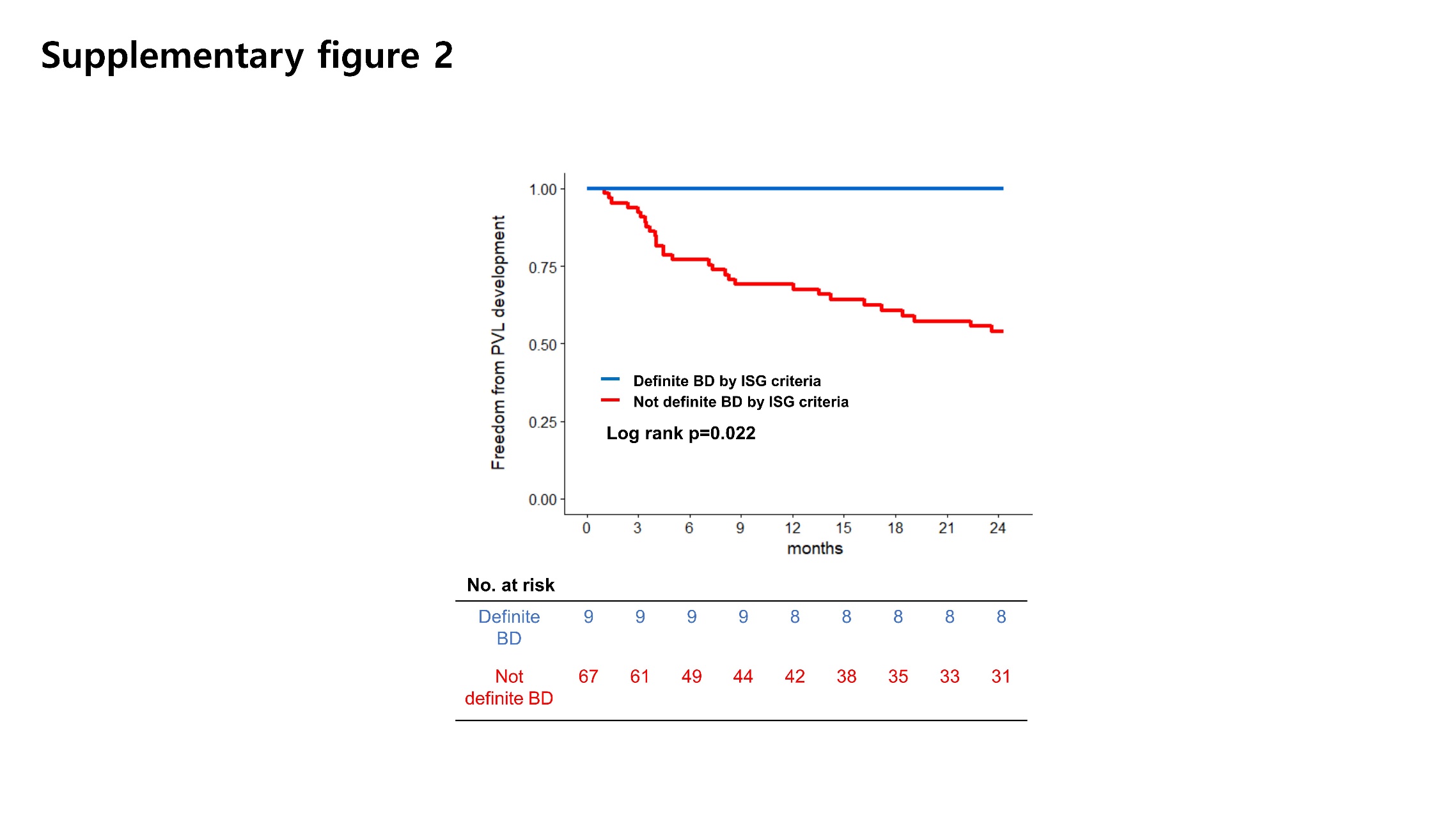


**Figure S3. Kaplan-Meier estimates of freedom from paravalvular leakage (PVL) in patients who received postoperative immunosuppressive therapy (IST), stratified by the use of preoperative (preop) IST**

Preoperative IST was not an independent predictor of less PVL development in patients who received postoperative IST.


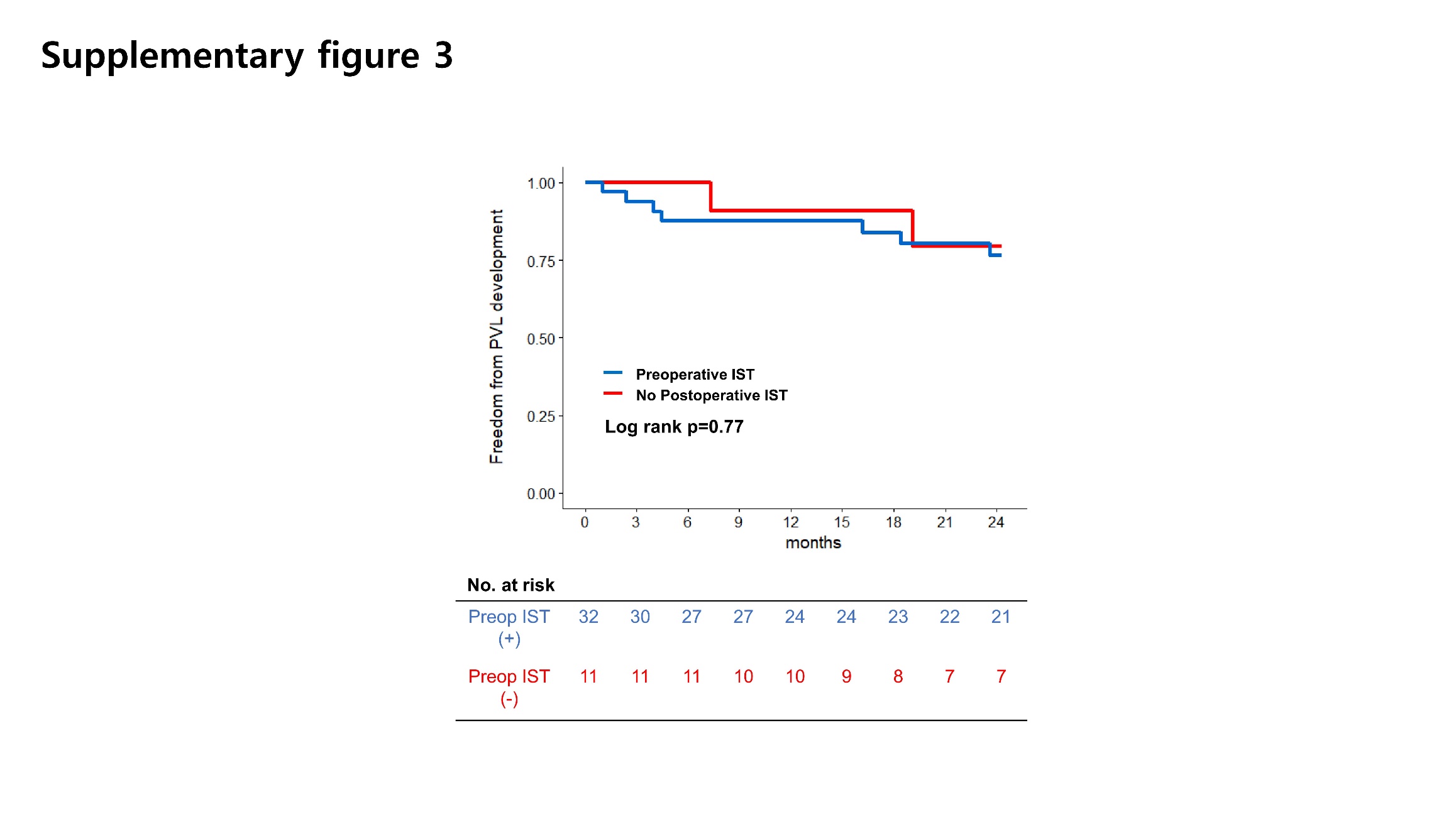

Supplement: Supplementary file 4 — Figure S1. Schematic diagram showing the selection process of the patient population. Figure S2. Kaplan-Meier estimates of freedom from paravalvular leakage (PVL) stratified by satisfaction of the International Study Group (ISG) criteria. Figure S3. Kaplan-Meier estimates of freedom from paravalvular leakage (PVL) in patients who received postoperative immunosuppressive therapy (IST), stratified by the use of preoperative (preop) IST. (DOCX 378 kb) [file 13023_2019_1083_MOESM1_ESM.docx]
